# Thrombosed umbilical vein varix in newborn with congenital syphilis

**DOI:** 10.1590/0037-8682-0406-2023

**Published:** 2023-10-13

**Authors:** Merve Erkan, İpek Güney Varal

**Affiliations:** 1 University of Health Sciences, Bursa Yuksek Ihtisas Training and Research Hospital, Department of Radiology, Bursa, Turkey.; 2 University of Health Sciences, Bursa Yuksek Ihtisas Training and Research Hospital, Department of Pediatrics, Division of Neonatology, Bursa, Turkey.

**FIGURE 1:**
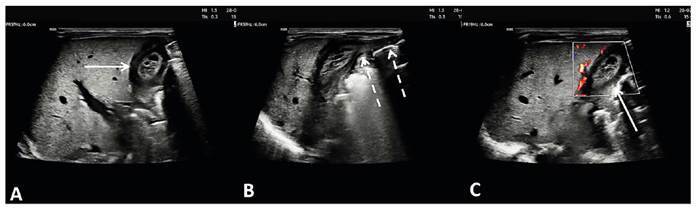
(A-B) Sonographic examination showing a heterogeneous partially solid and cystic tubular mass (arrow) arising from the left lobe of the liver to the umbilicus along the tract of the umbilical vein (interrupted arrow). (C) No flow was detected in the lesion on Doppler US (arrow).

A 30-year-old woman with syphilis during pregnancy gave birth to a preterm neonate who was admitted to the neonatal intensive care unit. The mother refused treatment for syphilis during pregnancy. After newborn screening, which identified positive Venereal Disease Research Laboratory (VDRL), treatment for congenital syphilis was initiated with crystalline penicillin for ten days. Cholestasis developed on postnatal day 6 (total bilirubin: 12.7 mg/dL, direct bilirubin: 7.6 mg/dL). Ultrasound (US) revealed a heterogeneous, partially solid, cystic tubular mass arising from the left lobe of the liver to the umbilicus along the tract of the umbilical vein (Figure 1A-B ). No flow was detected in the lesion on Doppler US (Figure 1C). A diagnosis of a thrombosed umbilical vein varix (UVV) was established.

UVV is the focal dilatation of the umbilical vein. The incidence is 0.4-1.1/1000. It is a rare anomaly prenatally diagnosed by identifying a hypoanechoic elongated mass between the fetal abdominal wall and the inferior edge of the liver with internal flow on Doppler US^1^. Thrombosis is a possible complication of UVV due to blood flow turbulence within dilated vessels. It can be diagnosed prenatally or during the neonatal period and can ultimately lead to fetal disseminated intravascular coagulation and fetal demise^2^. Although a few cases of thrombosed UVV in the prenatal period have been described in the literature^3^, it is an unusual diagnosis during the postnatal period. Awareness of this entity and imaging findings can aid in its diagnosis.
